# Anti‐TIF‐1γ dermatomyositis: Erythematous eruption with weakness and normal creatine kinase

**DOI:** 10.1002/ski2.366

**Published:** 2024-03-11

**Authors:** Lucy Mackie, Nilukshi Wijesuriya, Ashok Iyer

**Affiliations:** ^1^ Department of Medicine Croydon University Hospital London UK; ^2^ Department of Pathology St George's Hospital London UK

## Abstract

We describe a case of a previously well 76‐year‐old woman who presented with 9 months of a generalised pruritic rash alongside fatigue, weight‐loss and a symmetrical weakness of the legs.

## Graphical abstract

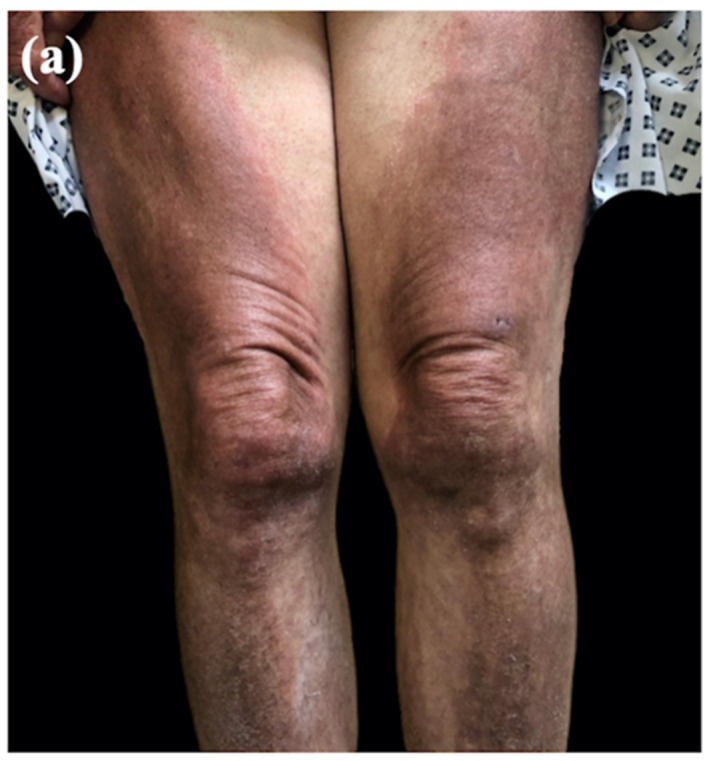

## 1 CLINICAL FINDINGS

A 76‐year‐old woman of Afro‐Caribbean descent presented with 9 months of a generalised pruritic rash alongside fatigue, weakness and weight‐loss. She had a history of septic arthritis and was taking colecalciferol. She had become largely bed‐bound, having previously been fully independent. Systems review revealed no other symptoms.

**FIGURE 1:**
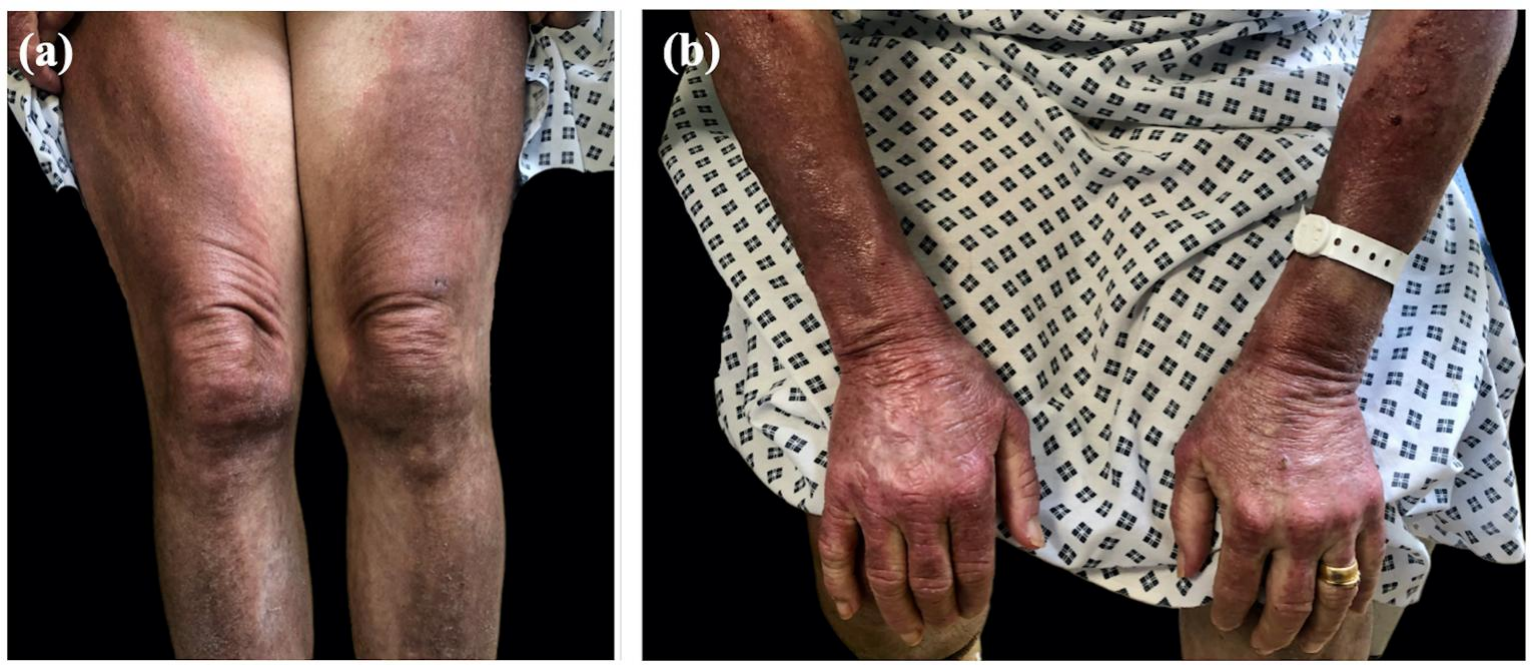
Panels (a, b) show sharply demarcated extensive erythema on the extensor surface of limbs including the metacarpophalangeal joints of the hand, with photosensitive sparing of the watch strap.

Examination showed widespread erythematous patches with superficial scaling especially affecting the face, chest, abdomen and extensor surfaces of the limbs. They were more severe and sharply demarcated on photo‐distributed areas, such as the upper chest and dorsum of the hands (Figure 1). She had generalised non‐scarring hair‐loss, ragged cuticles and prominent nailfold capillaries. Proximal leg power was 4/5.

Bloods showed slightly raised erythrocyte sedimentation rate (55 mm/hr) and C‐reactive protein (41 mg/L). Renal and liver function, creatine kinase (CK) and calcium were normal. An autoantibody screen was positive for anti‐transcriptional intermediary factor‐1γ (anti‐TIF‐1γ). Magnetic resonance imaging (MRI) of thighs showed myopathic features. Computerised tomography (CT) and positron emission tomography scans revealed an enlarged subcarinal lymph node with no primary lesion; biopsy of this suggested metastatic adenocarcinoma.

**FIGURE 2:**
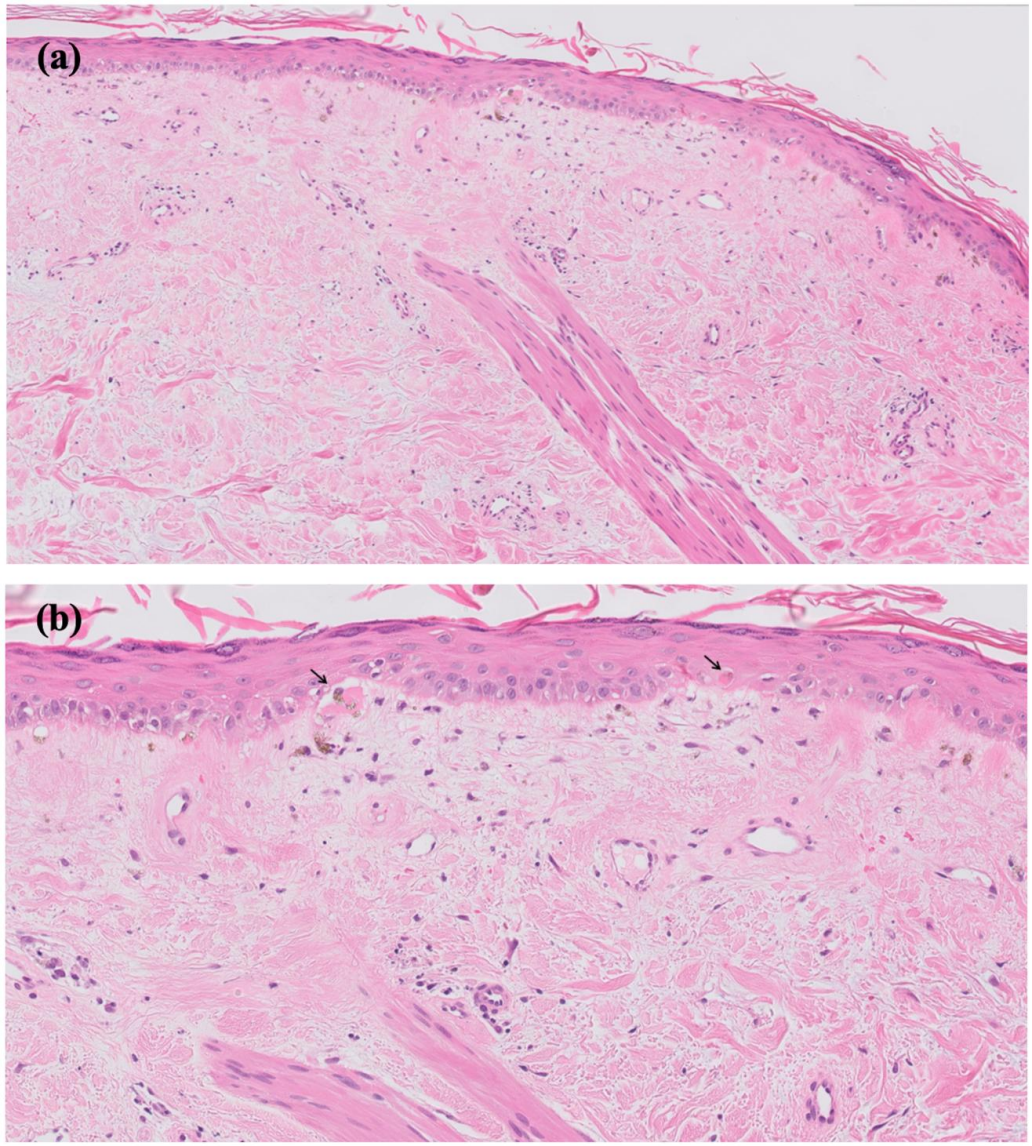
(a) Features of a pauci‐inflammatory interface dermatitis with changes limited to the upper dermis and (b) basal cell vacuolar degeneration with melanin pigment incontinence and scattered necrotic keratinocytes (arrows). 4 mm punch biopsy left thigh, haematoxylin and eosin, original magnification (a) 10× and (b) 20×.

Histopathology showed features of a pauci‐inflammatory interface dermatitis with compact hyperkeratosis (Figure 2). There was basal cell vacuolar degeneration, patchy pigment incontinence, occasional collections of necrotic keratinocytes and a mild perivascular lymphohistiocytic infiltrate (Figure 2). There was no evidence of a neutrophilic infiltrate or frank vasculitis. Direct immunofluorescence showed globular deposits of IgA and IgM and strong linear positivity of fibrinogen along the basement membrane zone.

This was diagnosed as paraneoplastic anti‐TIF‐1γ dermatomyositis (DM).

## 2 DISCUSSION

Dermatomyositis is a rare systemic autoimmune condition with characteristic cutaneous lesions and muscle inflammation. It is one of the idiopathic inflammatory myopathies, with the DM subgroup identified in 1863.^1^ It is more common in females (2:1 F:M), with peak incidence in middle age.^2^ There is also a juvenile onset form.

Pathogenesis is multifactorial, with autoimmune damage against endomysial capillary endothelium.^1^ Both genetic and environmental factors are likely involved. A number of myositis‐specific auto‐antibodies (MSAs) have been discovered, including anti‐Mi‐2, anti‐MDA‐5, anti‐NXP‐2, anti‐SAE and anti‐TIF‐1. Each MSA subgroup has increasingly understood characteristics, such as an association between anti‐MDA‐5 and interstitial lung disease (ILD).^2^ It is postulated that antibody‐associated clinical phenotypes will be incorporated into diagnostic criteria for DM, and will be used increasingly to risk‐stratify and manage patients.^2^ Antibodies to the anti‐TIF tumour‐suppressor protein subunits (1γ, 1α, 1β) were identified in 2006 and are found in 18%–23% of DM patients.^2^ Anti‐TIF‐1γ is more common than anti‐TIF‐1β, while anti‐TIF‐1α always co‐exists with the former.^3^

Pathognomonic dermatological features of DM include a heliotrope rash (purple discolouration of the eyelids), Gottrons papules (purple papules over the small joints of the hands), Gottrons sign (erythema over the backs of the hands, elbows and knees) and the shawl sign (erythema over the neck and shoulders).^2^ Cutaneous findings specific to anti‐TIF‐1 DM include a more severe dark‐red photosensitive rash, nail fold telangiectasia, pruritus, and unique psoriaform lesions and poikiloderma (skin atrophy with pigment changes and telangiectasia).^1^, ^2^, ^3^, ^4^, ^5^ Our patient demonstrates the pruritic rash, Gottrons sign, shawl sign and nail fold changes. Other features of anti‐TIF‐1 DM include mild myositis, dysphagia and high‐risk of malignancy. Associated malignancy has been reported in up to 65% of patients in some studies, although others describe much lower figures.^2^ An association is particularly seen with anti‐TIF‐1γ and anti‐TIF‐1α; risk is also increased in anti‐NXP2 DM.^2^, ^3^ The most frequently reported malignancies are ovarian, breast, lung, gastric, colorectal and lymphoma.^5^ In our patient a primary lesion was not found.

Myositis in DM normally involves proximal muscles with symmetrical sub‐acute weakness, fatigue and raised CK. Up to 20% of patients have clinically‐amyopathic DM, with no clinical muscle disease.^6^ This is often mis‐diagnosed as subacute lupus erythematous, which also has overlapping histopathological findings.^7^ Our patient had disabling clinical weakness but a normal CK, complicating diagnosis. Literature describes that CK can be normal in active disease, including in a case report with MDA5 and SAE1 antibodies, and a case series of anti‐TIF‐1β patients shows normal CK levels with mild muscle weakness.^3^, ^8^

The most recent diagnostic criteria are the 2017 EULAR criteria, with investigations including muscle enzymes, autoantibodies, skin/muscle biopsy, electromyography and MRI.^9^ Screening for malignancy is important, with dermatosis leading to diagnosis in this case. Other investigations may include electrocardiogram and echocardiogram for cardiac involvement, barium swallow for dysphagia, and CT chest and pulmonary function tests for ILD.

Our patient was treated with mometasone 0.1% ointment, tacrolimus 0.1% ointment and 120 mg fexofenadine before receiving 3 doses of pulsed IV methylprednisolone and a weaning regimen of oral prednisolone (from 40 to 10 mg per day). She also underwent radiotherapy. This improved weakness but not the rash. Management is often challenging and many patients require lifelong treatment. Steroid sparing agents can be considered, such as methotrexate or azathioprine. Intravenous immunoglobulin has been used for some patients.^10^ Management of malignancy is key in malignancy‐associated cases, although a case series describes some anti‐TIF‐1γ patients with a lack of remission despite cure of malignancy.^11^ Supportive care is important with photo‐protection, antipruritics and physical and occupational therapy.

This case illustrates the importance of recognising cutaneous features of DM with normal CK, and the diagnostic delay and disability that can occur. It highlights the severe erythema and malignancy associated with anti‐TIF‐1γ DM. It is important that patients are not treated as erythrodermic psoriasis with phototherapy, as this worsens DM, and are screened for malignancy.

## CONFLICT OF INTEREST STATEMENT

The authors declare no conflicts of interest.

## AUTHOR CONTRIBUTIONS

**Lucy Mackie**: Conceptualization (lead); data curation (lead); writing—original draft (lead); writing—review and editing (lead). **Nilukshi Wijesuriya**: Data curation (supporting); supervision (supporting); writing—review and editing (supporting). **Ashok Iyer**: Conceptualization (supporting); data curation (supporting); supervision (lead); writing—review and editing (supporting).

## ETHICS STATEMENT

Not applicable. Informed consent was obtained from the patient presented.

## Data Availability

Data sharing is not applicable to this article as no new data were created or analyzed in this study.
